# Validation of Psychophysiological Measures for Caffeine Oral Films Characterization by Machine Learning Approaches

**DOI:** 10.3390/bioengineering9030114

**Published:** 2022-03-11

**Authors:** Patrícia Batista, Pedro Miguel Rodrigues, Miguel Ferreira, Ana Moreno, Gabriel Silva, Marco Alves, Manuela Pintado, Patrícia Oliveira-Silva

**Affiliations:** 1 HNL/CEDH—Human Neurobehavioural Laboratory/Research Centre for Human Development, Universidade Católica Portuguesa, Rua de Diogo Botelho, 1327, 4169-005 Porto, Portugal; miguel.m.f_18@hotmail.com (M.F.); amoreno@ucp.pt (A.M.); posilva@ucp.pt (P.O.-S.); 2 CBQF—Centro de Biotecnologia e Química Fina—Laboratório Associado, Escola Superior de Biotecnologia, Universidade Católica Portuguesa, Rua de Diogo Botelho, 1327, 4169-005 Porto, Portugal; g.arsilva@hotmail.com (G.S.); marcoalves1996@hotmail.com (M.A.); mpintado@ucp.pt (M.P.)

**Keywords:** oral films, delivery systems, caffeine, electrodermal activity, electrocardiogram, respiratory activity

## Abstract

(1) Background: The oral films are a new delivery system that can carry several molecules, such as neuromodulator molecules, including caffeine. These delivery systems have been developed and characterized by pharmacokinetics assays. However, new methodologies, such as psychophysiological measures, can complement their characterization. This study presents a new protocol with psychophysiological parameters to characterize the oral film delivery systems based on a caffeine model. (2) Methods: Thirteen volunteers (61.5% females and 38.5% males) consumed caffeine oral films and placebo oral films (in different moments and without knowing the product). Electrocardiogram (ECG), electrodermal (EDA), and respiratory frequency (RF) data were monitored for 45 min. For the data analysis, the MATLAB environment was used to develop the analysis program. The ECG, EDA, and RF signals were digitally filtered and processed, using a windowing process, for feature extraction and an energy mean value for 5 min segments. Then, the data were computed and presented to the entries of a set of Machine Learning algorithms. Finally, a data statistical analysis was carried out using SPSS. (3) Results: Compared with placebo, caffeine oral films led to a significant increase in power energy in the signal spectrum of heart rate, skin conductance, and respiratory activity. In addition, the ECG time-series power energy activity revealed a better capacity to detect caffeine activity over time than the other physiological modalities. There was no significant change for the female or male gender. (4) Conclusions: The protocol developed, and the psychophysiological methodology used to characterize the delivery system profile were efficient to characterize the drug delivery profile of the caffeine. This is a non-invasive, cheap, and easy method to apply, can be used to determine the neuromodulator drugs delivery profile, and can be implemented in the future.

## 1. Introduction

Oral films (OF) or orodispersible films have received increasing attention from researchers and the pharmaceutical and nutraceutical market, as potential innovative drug delivery systems [1]. The firsts OF were approved in 2010 and were defined by the American Food and Drug Administration (FDA) as including active pharmaceutical ingredients with flexible properties with a quick dissolution or disintegration when in contact with saliva [2]. There are some advantages compared with traditional solid (e.g., tablet, capsule) and liquid dosage forms. These delivery systems present some benefits, including easy administration, convenience, not needing water, rapid disintegration, higher bioavailability and the fact that the molecule being carried can be absorbed through the buccal mucosa and can directly enter the systemic circulation, avoiding the first-pass metabolism [3,4,5].

Several molecules can be incorporated into these delivery systems, such as benzocaine, phenylephrine, sildenafil, ondansetron, chlorhexidine, captopril, as well as neuromodulator molecules (donepezil, tianeptine, zaleplon, paracetamol, buspirone hydrochloride, aripiprazole, caffeine) [2,6,7,8,9,10,11]. These active molecules can be carried in dispersed or loaded form into microparticles or nanoparticles depending on the physicochemical properties of the drug and the desired release pattern [1,9,12,13,14,15].

However, the current legislation about OF development and production is not clear [1,16]. European Pharmacopeia presents a brief description of the dosage form and also mentions that OF should have an adequate drug release and mechanical strength. Still, most of the other OF quality attributes are yet to be categorically defined [1,16]. To clarify this point, it is important that the regulatory entities step out the means of quality standards for OF evaluation to ensure patient or consumer safety.

Many of these products are considered nutraceuticals and are already on the market (e.g., caffeine films). Caffeine is a widely consumed psychostimulant compound. As an example, it is estimated that more than 80% of the world’s population consume caffeine [17]. Caffeine is consumed as an ingredient incorporated in several products, and more recently, this substance has been incorporated into oral films [18]. This neuromodulator molecule has an attractive potential because it is an ergogenic aid, improves various cognitive and/or bodily functions, including alert and concentration state, energy, mental and physical performance [19]. However, while a plethora of research exists proving caffeine’s benefit to performance, there are also secondary effects [20]. For instance, because caffeine is a molecule rapidly absorbed in the gut (99% in 45 min after intake), leading to the need for constant intake along the day to maintain the expected effects [21], the typical caffeine intake through a cup of coffee is not compatible with highly time-demanding professions. Additionally, some sensitive individuals may experience a significant increase in nervousness, anxiety, tension, irritability, tremor, perspiration, palpitations, dizziness, and even headache if they consume high doses of caffeine. The OF can carry several neuromodulator molecules that stimulate the Autonomic Nervous System (ANS). While the electrodermal measures reflect the eccrine sweat gland activity in response to stress or emotional arousal mediated by the sympathetic branch of the ANS; the cardiac measures reflect a synergy between the sympathetic and the parasympathetic branches, being highly sensitive to a variety of psychological or physiological conditions, such as the regulation of emotions or cognitive effort [22]. Finally, the respiratory measures have emerged as an index of anxiety, also changing during focused attention periods or cognitive demanding tasks involving an increase in the anxiety level [23].

The premise that psychophysiological measures, such as heart rate, electrodermal activity, and respiratory activity, correlate with the cognitive and affective functions [24], can offer an essential insight into cognitive and internal emotional experiences that are not available by assessing self-report. Besides involving a cost-efficient technique, the psychophysiological measures are also easier to monitor.

The psychophysiological measures are important parameters to characterize neuromodulator molecule delivery systems and can complement the guidelines to ensure the safety and quality of this new delivery system. The psychophysiological characterization integrates data from the cognitive activity of an individual (e.g., monitoring cognitive processes, such as attention) with the evaluation of physiological indexes, which are connected to the ANS [25]. There are several psychophysiological approaches based on the technological advancement of instruments such as functional Magnetic Resonance Imaging, magnetoencephalography, and Emission Tomography techniques, but these are expensive techniques, and difficult to access. However, peripheral psychophysiological measurements, such as the skin conductance response, electrocardiogram, electromyography, electroencephalogram, blood pressure, respiration, pupillary diameter and eye-tracking are relatively simple to perform, non-invasive, and have accurate real-time results [19].

Although caffeine has been widely studied (its pharmacokinetics and dynamics profile are already well known), its other physiological effects, especially when consumed through OF, deserves further study. For this reason, we use this molecule when incorporated into OF to characterize its effect at the level of peripheral psychophysiological parameters.

Thus, we aimed at developing and validating a new protocol to improve OF characterization by developing an understanding of the psychophysiological impact of the caffeine embedded in the OF, namely through its impact on the electrodermal, cardiac and respiratory systems. The psychophysiological characterization of these OF is of great importance for the overall quality and efficacy of the product before scale-up, because this procedure provides evidence of the sustained release of caffeine which can act along the day without the well-known side effects.

## 2. Methods

### 2.1. Participants

A stratified random sample with thirteen participants was performed in this study. Participants were healthy volunteers with an age range from 18 to 46 years old who met the inclusion criteria (older than 18 years old and consumer of coffee or other caffeinated product on a daily basis). Participants were excluded if they had any cardiac disease or implant; substance abuse disorders; used medication that affects cardiac activity or endocrine function (e.g., beta-blockers); had any physical or mental conditions that might affect the physiological measurements; were pregnant, breastfeeding or smokers. Sociodemographic data were obtained using an online questionnaire. The data were collected between 3 May 2019 and 13 January 2020.

The basic information for the participants was as follows. The mean age was 24.15 years old with a standard deviation (SD) of 7.71 years and consisting of 61.5% females and 38.5% males. All participants were healthier and had no history of cardiovascular diseases. Additionally, the present study was conducted according to the Helsinki declaration and has been previously approved by the Internal Research Ethics Committee. All participants provided their written informed consent before the first session of the study.

### 2.2. Data Collection

Concerning the self-report data, a questionnaire was applied that included a sociodemographic section (e.g., sex, age, medical or psychiatric illness, caffeine, and tobacco use).

For the psychophysiological measures, a continuous measure was performed for each participant from the baseline, at a sampling rate of 1000 Hz. The BIOPAC MP160 and the AcqKnowledge software 5.0 of December 2017 (by BIOPAC Systems Inc., Santa Barbara, CA, USA) were used as the data acquisition system for this study, with the following three modules: (1) the ECG100C cardiac activity amplifier measures; (2) the EDA100C electrodermal activity amplifier to measure the skin conductance level which varies with eccrine sweat gland activity in response to stress or emotional arousal; and (3) the RSP100C respiratory activity amplifier to measure the expansion and contraction of the abdominal or thoracic region while breathing.

### 2.3. Procedures

This study used an intra-subject design which consisted of two laboratory sessions held on two consecutive days, both during the morning. After going through an eligibility screening, participants were appointed to two laboratory visits where the study took place in a well-controlled laboratory environment. Days before the laboratory appointment, participants were requested to keep the same caffeine intake pattern until the data collection, but to avoid any psychostimulants such as chocolate, cola, or even coffee two hours prior to the session.

In the first visit, after explaining the study’s procedures and providing their consent to participate in research, participants were randomly assigned to one of the two conditions for that day, of either OF with caffeine or OF without caffeine (these OFs were produced in the Centro de Biotecnologia e Química Fina in the Universidade Católica Portuguesa by an experienced research team in the field) [13,26,27]. In the second visit, they had the other missing condition. The randomization for the conditions’ sequence was obtained using a computer-generated code via the website: http://www.randomization.com (accessed on 22 February 2020). Consequently, the procedure was fully blinded for the participants and the researcher assistants in charge of the data collection.

**Figure 1:**
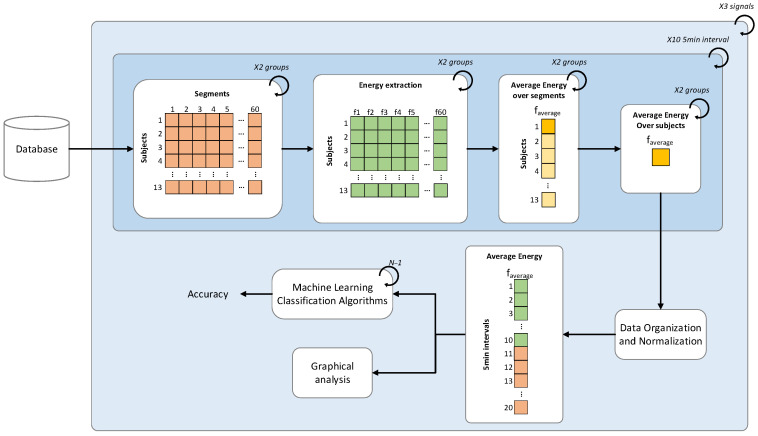
The Time Signal Analysis methodology.

After the completion of the questionnaires, the lab equipment was set up and participants were asked to remain relaxed for some minutes to ensure their habituation to the lab environment (please see Figure 1 for an overview of the study procedure).

The experimental procedure consisted of the following four phases: (i) welcoming, informed consent, instructions, application of the questionnaire and equipment setup; (ii) baseline task; (iii) oral film intake; and (iv) monitoring phase, during which the experimental tasks were administered while the psychophysiological signals were recorded.

Furthermore, after 5 min of resting in the sitting position, electrodes were placed on the participants in suitable positions to collect the psychophysiological measures. The participants completed a socio-demographic questionnaire. Subsequently, the baseline task was performed.

Concerning the baseline task which was performed before caffeine intake, we obtained baseline values for each psychophysiological modality used in this study. For this task, participants performed an approximately 15 min task, requiring minimal demanding effort, in which they were asked to examine and rate whether pairs of pictures at 1 min intervals were different or equal during 5 min. Then, they performed a brief version of the behavioural task to analyze the cognitive performance level prior to the caffeine intake (see below). The low-arousal images were taken from the International Affective Picture System (IAPS) database, according to the validation for the Portuguese population [28]. A 5 s fixation cross was displayed between each pair of images.

Then, the next task was OF administration, according to the condition randomly appointed for that subject in the visit (i.e., OF with caffeine or OF without caffeine). In other words, participants received an OF with caffeine in the 1st day and an OF without caffeine in the 2nd day, or vice versa. At the time of consumption, the participant places the OF into the oral cavity and the film disintegration occurs in about 30 s without the need for water.

For the monitoring phase, after the OF consumption, participants were asked to:

- Rate their alertness using the Visual Analog Scales (VAS), choosing among “alert/able to concentrate”, “anxious”, “energetic”, “feel confident”, “irritable”, “jittery/nervous”, “sleepy”, and “talkative” [29,30];
- Perform the Attentional Network Test (ANT), a computer-based test to measure participants’ performance in 3 separate components of attention: alerting, orienting, and executive control [31,32];
- Perform the Test of variables of attention (T.O.V.A.), which is a continuous performance test that assesses attention and impulsivity [33].

During this monitoring time (baseline and monitoring phases), the participants were also monitored using the BIOPAC data acquisition system (Biopac System, Santa Barbara, CA, USA) in conjunction with the Acknowledge software 3.6.7 (Biopac Systems) to assess three psychophysiological signals: electrocardiogram (ECG), respiratory frequency (RF) and electrodermal activity (EDA). The entire procedure lasted around 45 min (minimum of 36.8 min and maximum of 48.3 min).

### 2.4. Experimental Indicies

In this study, ECG, RF, and EDA data were collected from the same participants on two separate occasions, with no knowledge of which group they were assigned to. As a result of the two distinct instances, we were able to form two groups of thirteen participants each.

- ECG

The ECG was monitored with a standard electrode configuration (right clavicle and precordial site V6). Two disposable Ag-AgCl electrodes were used. ECG measurement was performed with a sampling frequency of 100 Hz using ECG100C unit and BIOPAC Acqknowledge acquisition software (Biopac System Inc., Santa Barbara, CA, USA) connected to a computer.

All of the ECG tests were performed in the morning hours at the same time of the day to avoid the diurnal variability in ECG parameters.

- RF

The RF signal was registered using a thoracic inductive plethysmography band to analyze the changes in the inhalation and exhalation periods. The results were collected using BIOPAC.

- EDA

EDA or galvanic skin response is a psychophysiological response that can be assessed by measuring changes in the electrical properties of the skin.

The electrodermal responses were measured using two standard 8 mm Ag/AgCl electrodes placed on the medial phalanges of the palm of the non-dominant hand of the participant after controlling for hydration and the temperature of the environment (21 °C). Participants were asked to wash their hands with neutral soap and water to diminish the impedance and to improve the quality of the signal, prior to the attachment of electrodes. In order to record the responses, these electrodes were connected to a BIOPAC.

### 2.5. Time Signal Analysis

The database was loaded in a MATLAB^®^ environment. All raw signals were digitally filtered. For EDA and RF signals, an FIR low-pass filter was applied with cut-off frequencies 1 Hz and 3 Hz, respectively, and for ECG signals, an FIR band-pass filter was used with cut-off frequencies of 1 and 30 Hz. Then, the amplitude of each signal was normalized according to,

(1) $$x\left(n\right)=\frac{x\left(n\right)}{\sum_{n=0}^{N-1}x^{2}\left(n\right)},$$

where *N* represents the signal size, and the mean value was removed. In each signal modality (ECG, EDA, RF), per subject, the full-band signal was split into segments of 5 min for analysis. From each segment, the signal power energy (*EN*) was computed every 5 s within a sliding rectangular windowing process as,

(2) $$EN=\overset{N}{\underset{n=1}{\sum }}{\left|x\left(n\right)\right|}^{2}.$$

where, *N* is the signal 5 s length.

Then, the 5 min signal power energy features distributions underwent an averaging process per group and a mean value representing the group mean power for each 5 min segment length was obtained. Then, the mean power energies distributions per group along the 50 min (10 points per group) have been normalized with z-score method for graphical analysis and to feed machine learning algorithms for groups discrimination. In the Machine learning algorithm phase, several binary classifiers, namely, decision trees (DT), support vector machines (SVM), k-nearest neighbors (KNN), logistic regression, discriminant analysis and X-ROC were used. In all cases, a leave-one-out cross-validation procedure is used, to verify the generalization capacity of the classifiers. The Classification learner toolbox provided by Matlab^®^ has been used for the propose. The Time Signal Analysis methodology is illustrated in the following Figure 1.

### 2.6. Statistical Analysis

The socio-demographic data analysis was carried out using SPSS version 24.0 (IBM Corporation, Armonk, NY, USA). The parameters have been organized as mean ± SD. A *p* < 0.05 was considered statistically significant. The behavioural data, such as the response times from the cognitive and mood tasks as well as from the physiological responses were also analyzed.

## 3. Results

Thirteen participants were screened, and socio-demographic characterization are presented in Table 1.

The sample was also characterized with respect to the pattern of the caffeine consumer profile (Table 2)

When participants were asked whether they consumed caffeine in other products, some said they did not consume caffeine in any other products 38.5%, 38.5% mentioned they consumed caffeine in tea and 15.4% in beverages.

### 3.1. ECG Activity

**Figure 2:**
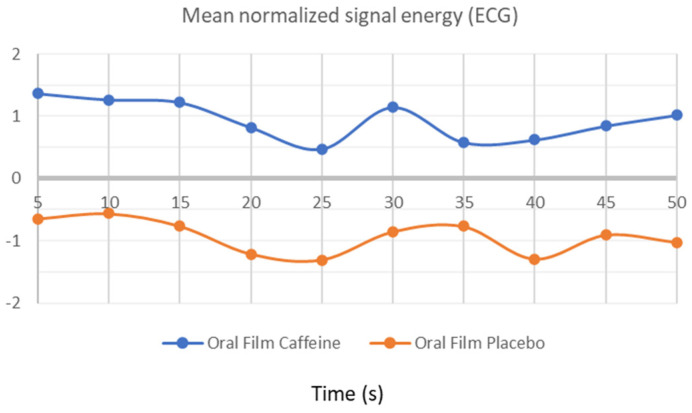
Energy signal obtained by electrocardiogram analysis, during OF consumption.

As can be seen in Figure 2, heart rate signal energy over 50 min was positively higher for participants who consumed caffeine OF. When comparing the energy signal of heartrate evaluation after consumption of OF with or without caffeine, a higher energy signal was found for OF with caffeine consumption as expected. However, the wave pattern remains with moderate slopes because these devices facilitate a gradual release of caffeine over time. Some caffeine included in these films was encapsulated into microparticles, which allows for a slower release profile; however, there is also some free caffeine content in the film.

### 3.2. EDA Activity

**Figure 3:**
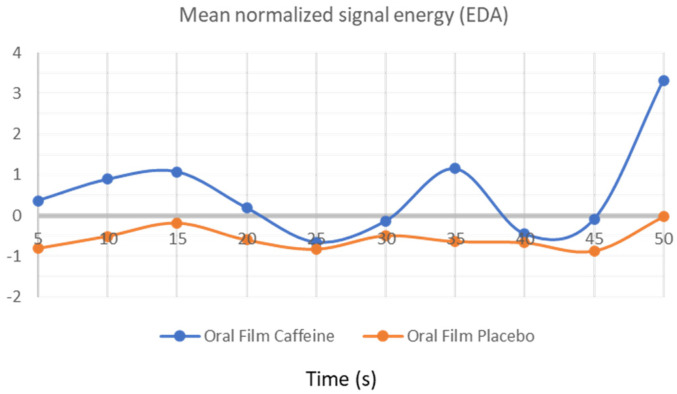
Electrodermal energy signal obtained after OF consumption.

Similar results were found when the electrodermal energy signal was analyzed. The electrodermal energy signal was tendentially higher for the caffeine OF consumption (Figure 3). However, because the lines in the EDA signal are much closer together, the energy differences between the two groups are not as substantial as they are in the ECG signal. In the last 5 min, we are able to see how the mean normalized signal energy increased in both groups, with the caffeine group having a significantly larger influence. This issue may be due to the electrodes being removed incorrectly at the end of the process, resulting in a very high energy noise peak.

### 3.3. Respiratory Frequency

**Figure 4:**
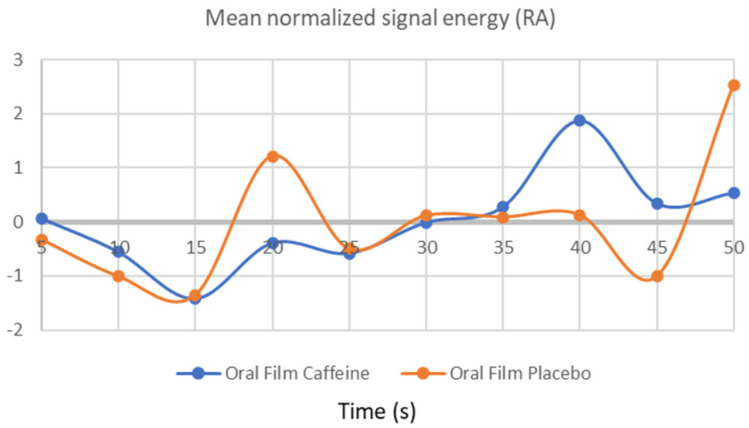
Respiratory frequency signals obtained after OF consumption.

The piecewise traces acquired for rising breath rate segments come from time (Figure 4).

### 3.4. Machine Learning Groups Discrimination

The accuracy of the discrimination results between the OF caffeine group and OF without caffeine groups are presented in Table 3. The optimal parameters used in each classifier are also indicated in Table 3.

As can be seen, the ECG time-series analysis is sufficient to detect the presence of caffeine, as outstanding results of 100% of discrimination accuracy were reached with all the classifiers used in this study. This was expected to happen due to the significant gap between the graphic lines in Figure 2. The discriminate accuracy across research groups was marginally worse for the other signal modalities than for the ECG. It was predictable that this would happen as the EDA graph lines were not as well separated as the ones in ECG graphic analysis, but they were still far enough apart to produce a very strong group discrimination accuracy of 85%. Finally, the RF would pose a challenge to classifiers due to the inflections points that are displayed along the graphic time series and how close the graphic lines are to each other over time. Thus, the used classifiers only achieved an accuracy of 60% for this modality. From all considered classifiers, Table 3 also shows that the XROC binary classifier had the best overall performance.

## 4. Discussion

Multiple studies have laid emphasis on the OF importance compared with traditional methods of drug delivery [7]. However, the guidelines for production and commercialization are not completely defined [16] and it is important to draw attention to this problem because there are already many products on the market, especially in nutraceuticals market and over-the-counter [3,19].

The use of non-invasive protocols that assure the characterization profile is interesting, and the psychophysiological measures can complement this characterization when the molecules carried are neuromodulator molecules. This protocol that we presented in the methods section, is an innovative protocol designed specifically to characterize OF that carry the neuromodulator molecule, such as caffeine. We selected the caffeine model because there is known in the literature and due to excessive consumption of this molecule worldwide [34].

Psychophysiological measures such as ECG, EDA, and RF are common parameters used to analyze the relationship between human behaviour and physiology. The psychophysiological measures are advantageous because they carry information about the physiological processes taking place at the time and are not dependent on the ability of self-evaluation and perception; can be assessed continuously and during the performance of other tasks; reflect autonomic regulation mechanisms that are not accessible to voluntary control; and are not distorted by social relationships and memory [35]. Their monitoring consists of the assessment of the activation and functioning level of the body [36]. In this way, these measures are presented as parameters capable of evaluating these new delivery systems that can carry caffeine, in order to assess the cognitive and mood effects that result from the release profile of this substance.

The ECG was able to obtain signals issued by the cardiovascular system. These signals are particularly interesting for psychophysiology because it is highly sensitive to neurological processes and psychological factors, as well as o the interaction between sympathetic and parasympathetic control. In our study, when analysing the ECG energy signal, we were able to see an increase in the energy signal with OF caffeine consumption compared with OF without caffeine, as well expected. The cardiac activity resulting from the biological effect of caffeine and the heart rate increase with caffeine consumption is known in the literature [20,37]. The data obtained corroborated this effect. However, the results showed the inexistence of accentuated peaks, showing controlled cardiac activity, due to the methodology of caffeine release by these devices—OF. This innovative delivery system with encapsulated caffeine allows for the gradual caffeine release over time, due to its increased bioavailability in the body, as reported in the literature [14,19].

So, the use of this psychophysiological measure included in this protocol is an excellent parameter to characterize the OF profile, as verified by the data obtained after the ECG time-series analysis. With several classifiers used, we obtained a higher discrimination accuracy for this parameter, with very satisfactory results.

In this study, an analysis of two other parameters well reported in the literature is also included. The incorporation of these three psychophysiological measures is extremely important because they are interconnected. The heart rate variability may vary in response to factors such as breathing rhythm, and therefore, it is important in this analysis. Respiratory frequency is closely related to social, emotional, and cognitive factors, and caffeine modulates the central mechanisms controlling breathing frequency as reported in the literature [36]. This information could be corroborated in this study because this measure allows for the verification of the increase in respiratory rate when the caffeine OF was consumed. However, the graph tracing was found to be very irregular, and the discrimination accuracy did not show a high excellence result for this parameter. Further studies should be carried out because this data did not ensure the expected efficiency for this psychophysiological measure in the OF characterisation. Additionally, the normalized energy may not be the best biomarker for this signal, and other metrics should be extracted and analyzed.

To complement the OF characterization, we included the EDA, the assessment of skin conductance (skin conductance level and skin conductance response), which is considered an indicator of psychophysiological activation. Similar to heart rate, EDA parameters have also been used indicators for arousal in physiological research [38]. EDA has been used in different studies, from the baseline evaluation of activation levels to the study of emotions and characterization of best performances [36,39]. The results obtained were very similar to those obtained for ECG, highlighting the most intense energetic signal during the consumption of caffeine OF. However, the discrimination accuracy was not as good as in ECG analysis, but still very satisfactory.

These psychophysiological measures were selected because they are multidimensional and multimodal approaches to investigate performance, integrating physiological measurements with behavioural and psychological data [36,38]. These psychophysiological measures also have the advantage of assessing, easy capturing, robustness, and the reflection of the sympathetic nervous system activity and the measurement is technically straightforward.

So, the aim of this study was to develop a new protocol by which to characterize the oral film’s profile and we obtained an interesting result with regard to their use. This is the first study in the literature demonstrating a new methodology to characterize the neuromodulator drugs delivery profile.

Some limitations are acknowledged in this study. Due to the small sample size, our results should be considered exploratory and not confirmative of our hypotheses. Future studies should include a large number of subjects. Another limitation is the lack of heterogeneity due to gender, meaning that other studies should try to balance the number of women and men in order to reproduce this study. Additionally, we applied only short-term measurements of cardiovascular parameters, also essential to the investigation of caffeine OF’s long-term physiological effects. Finally, we recommend that future studies compare these psychophysiological measures with other modalities, such as MRI, which is equally non-invasive but more accurate in terms of pharmacological agents’ pattern and mechanisms of action.

## 5. Conclusions

The current investigation is, to our knowledge, the first to characterize oral films using a psychophysiological protocol. By using a caffeine oral film, as a known neuromodulator molecule, we evaluated, via heart rate, breath rate and electrodermal activity, the caffeine delivery profile into the body.

The use of the psychophysiological measures can complement the oral film’s characterization of carrier neuromodulator molecules. Findings indicated higher energy levels when participants consumed caffeine OF. The excellent findings achieved in the ECG assessment stand out as the energy is well matched with the ECG signal. Other metrics, however, should be extracted for clearer inferences about the remaining signals, because the energy of these signals may be altered by numerous other biological events that have a greater effect than the consumption of the OF.

This new protocol is easy to execute, non-invasive and showed clear evidence of the caffeine profile into the body after the consumption of caffeine oral films.

## Figures and Tables

**Table 1:** Socio-demographic characteristics of the participants (*n* = 13).

	Continuous Measure	Min	Max	Mean		SD
Age		18	46	24.15		7.71
	**Categorical Measure**				**%**	
Gender						
Females					61.5	
Males					38.5	
Marital status						
Single					92.3	
Married/In a relationship					0	
Divorced/separated					7.7	
Widower					0	
Education levels						
Elementary School					7.7	
Secondary					61.5	
Higher education (degree)					23.1	
Higher education (master’s, doctorate and post-doc)					7.7	
Profession						
Students					84.6	
Other profession					15.4	

**Table 2:** Caffeine consumption profile (*n* = 13).

	Categorical Measure	%
Enjoy coffee		
Yes		84.6
No		15.4
Frequency of coffee drinking per day		
Up to once a day		38.5
2 times a day		7.7
2 to 3 times a day		23.1
More than 3 times a day		7.7
Rarely		15.4
A few times a week		
Reasons for drinking coffee		
Wake up		15.4
Socially		7.7
Health		7.7
Several (no specific)		69.2

**Table 3:** Accuracy results of OF caffeine and OF without caffeine groups.

Classifier	Optimal Parameters	Accuracy (%)		
		ECG	EDA	RA
Decision Trees				
Fine Tree	Maximum number of splits = 150	100	70	20
Medium Tree	Maximum number of splits = 150	100	70	20
SVMs				
Linear Kernel	Box constraint level = 5	100	80	0
Quadratic Kernel	Box constraint level = 3	100	70	40
Cubic Kernel	Box constraint level = 2	100	70	50
Nearest Neighbor Classifiers				
Cosine KNN	Number of neighbors = 3	100	80	35
Cubic KNN	Number of neighbors = 3	100	70	50
Discriminant Analysis				
Linear	Covariance structure: Full	100	75	0
Logistic	Covariance structure: Full	100	80	0
Quadratic	Covariance structure: Full	100	75	40
XROC	-	100	85	60

## Data Availability

Not applicable.
